# Iodine and Thyroid Dysfunction in Ageing: Nutritional, Pharmacologic, and Microbial Modifiers in Older Adults

**DOI:** 10.3390/geriatrics11010012

**Published:** 2026-01-26

**Authors:** Corina-Aurelia Zugravu, Marta Petre, Ciprian Constantin

**Affiliations:** 1 Department of Hygiene and Nutrition, “Carol Davila” University of Medicine and Pharmacy, 050463 Bucharest, Romania; corina.zugravu@umfcd.ro; 2 National Institute of Public Health, Dr. Leonte Street 1-3, 050463 Bucharest, Romania; 3 Research Metabolism Center, Faculty of Medicine, Titu Maiorescu University, 76 Al I Cuza Blvd, 011053 Bucharest, Romania; 4 “Carol Davila” Emergency Hospital, 88 M. Vulcanescu Street, 010825 Bucharest, Romania

**Keywords:** ageing, elderly, Geriatrics, gut microbiota/dysbiosis, polypharmacy, hypothyroidism, Wolff–Chaikoff effect, iodine intake

## Abstract

**Background**: Ageing profoundly alters endocrine regulation and nutrient metabolism, predisposing older adults to thyroid dysfunction. Iodine, an essential micronutrient, lies at the center of this vulnerability due to its narrow physiological range and multiple interactions with nutrition, medications, renal function, and, presumably, gut microbiota. **Objective**: This narrative review integrates evidence on how ageing modifies iodine–thyroid homeostasis, emphasizing the roles of dietary intake, pharmacologic exposures, microbiota composition, and age-related metabolic alterations that influence iodine handling and thyroid hormone economy. **Main Findings**: Physiological ageing reduces renal iodine clearance, thyroidal reserve, and peripheral hormone conversion, while chronic inflammation and multimorbidity increase susceptibility to both iodine deficiency and excess. Polypharmacy, including amiodarone, lithium, and proton pump inhibitors, further destabilizes thyroid function. Age-related dysbiosis may impair micronutrient absorption and immune tolerance, linking gut ecology to thyroid autoimmunity. The gut microbiota may influence thyroid function through immune and metabolic pathways, although current evidence in older adults remains limited. Together, these factors shift the balance between iodine intake and utilization, heightening the risk of subclinical or overt hypothyroidism in older adults. **Conclusions**: Overall, variations in iodine intake emerge as one of the main determinants of thyroid dysfunction in ageing with nutritional, pharmacologic, and other modifiers primarily influencing iodine-related thyroid vulnerability. The adoption of age-adjusted thyroid reference ranges and preventive monitoring can reduce overtreatment and improve metabolic resilience in later life.

## 1. Introduction

Ageing profoundly influences endocrine and metabolic homeostasis, and the thyroid gland is particularly sensitive to these changes. Adequate thyroid hormone synthesis and metabolism require the coordinated availability of several essential micronutrients, including iodine, selenium, iron, and zinc. Among these, iodine plays a unique and non-redundant role as the structural component of thyroid hormones and represents the principal nutritional determinant of thyroid dysfunction in ageing populations. Both insufficient and excessive iodine intake may disrupt thyroid homeostasis in older adults, with iodine deficiency remaining the predominant global risk factor for hypothyroidism in ageing populations. Ageing introduces physiological and metabolic changes that modify iodine–thyroid homeostasis, increasing vulnerability to subtle or overt thyroid disorders [1]. The relationship between iodine intake and thyroid dysfunction follows a U-shaped curve: iodine deficiency predisposes to goiter and hypothyroidism, whereas iodine excess or acute exposure may induce hypothyroidism or, less frequently, hyperthyroidism in susceptible older individuals [2]. By 2050, over 21% of the global population will be aged 60 years or older. This demographic is particularly vulnerable due to altered thyroid physiology [3,4] multimorbidity, and frequent drug exposures.

Over recent decades, widespread adoption of mandatory or voluntary salt iodization (USI), together with improvements in animal feed supplementation, food-processing standards, and agricultural practices, has markedly reduced endemic goiter and severe developmental consequences of iodine deficiency in many regions [5,6]. USI has dramatically reduced endemic goiter and iodine deficiency disorders, particularly in historically deficient regions [7,8]. Nevertheless, global iodine nutrition has become increasingly heterogeneous: while many populations now maintain adequate iodine status, significant pockets of persistent deficiency remain, especially among marginalized rural and remote communities, and paradoxically, iodine excess (either episodic or chronic) has emerged in certain settings due to high environmental iodine exposure, supplements, seaweed consumption, or iatrogenic sources [9,10].

These trends underscore the need for public health strategies that move beyond a binary ‘deficiency vs. adequacy’ framework toward dynamic, context-sensitive monitoring and fine-tuned interventions tailored to population and subpopulation needs [11].

Sources of iodine and determinants of intake are multiple and variable. The principal population-level intervention remains iodized salt, but dietary sources (seafood and seaweed, dairy, and iodophor-treated foodstuffs), fortification of animal feeds (which raises iodine content of milk and meat) [12], multivitamin/mineral supplements, and iatrogenic sources (iodinated contrast media, amiodarone, topical iodophors, etc.) all contribute to individual exposure. Geochemical factors (soil iodine content), inland versus coastal ecology [13], and food-system changes (increasing consumption of processed foods often made with non-iodized industrial salt) modify the effectiveness of USI and create regional variability.

Population iodine status is most commonly assessed by the median urinary iodine concentration (UIC) in spot urine samples [14]. The World Health Organization (WHO) benchmarks define adequacy in non-pregnant population groups by a median UIC of 100–199 µg/L (with 150–249 µg/L indicating adequacy in pregnancy) [15].

Despite these monitoring tools, programmatic challenges persist. First, UIC reflects recent intake and is highly variable for each individual; single spot measurements are meaningful only when aggregated as population medians, and interpreting borderline values therefore requires careful contextualization. Second, the global push toward dietary salt reduction for cardiovascular prevention complicates iodine prophylaxis: lower consumption of household salt reduces a major vehicle for iodine delivery unless iodization levels and fortification strategies are adapted and industrial food producers use iodized salt. Third, the decentralization of food production and the rise in consumption of processed and restaurant foods—often produced with non-iodized industrial salt—can erode the coverage and impact of USI unless regulatory frameworks extend to industrial salt. Emerging risks of iodine excess are increasingly recognized and multifactorial. In some populations, chronic excess arises from high habitual intake of iodine-rich foods (notably certain seaweeds and supplements) [16,17] over-fortification of salt or foodstuffs [18] or environmental/clinical exposures that acutely raise intrathyroidal iodide (like iodinated contrast agents, amiodarone therapy, and repeated topical povidone–iodine application) [19,20]. Epidemiologic observations during and after salt-iodization programs in previously deficient regions have documented transient rises in autoimmune thyroiditis and subclinical hypothyroidism—phenomena attributed to increased iodination of thyroglobulin, greater oxidative stress within thyrocytes, and the unmasking of underlying autoimmune susceptibility [21,22,23].

At individual levels, the elderly may be relatively more susceptible to iodine-induced hypothyroidism because of diminished glandular reserve, altered renal clearance prolonging iodide exposure, and a higher prevalence of thyroid autoantibodies; conversely, certain coastal populations with traditional seaweed consumption maintain high habitual iodine intakes without overt disease. Also, thyroid malfunction is frequently linked in older adults with dyslipidemia, atherosclerosis, cognitive decline, neuromuscular dysfunction, sarcopenia, osteoporosis, and frailty [24].

This narrative review synthesizes current evidence on iodine nutrition and thyroid dysfunction in older adults, emphasizing the intersecting effects of ageing on thyroid physiology, pharmacologic exposures, nutrition, gastrointestinal function, and gut microbiota. The term “older adults” refers primarily to individuals aged ≥65 years, with specific consideration of very old populations (≥85 years) and centenarians, in whom age-related changes in thyroid hormone economy—such as higher serum TSH and lower FT3/FT4 ratios—are well documented. We highlight key mechanisms and epidemiological data linking these domains and identify knowledge gaps relevant to clinical and public health practice. The specific aim of the review is to integrate these perspectives to clarify how ageing modifies iodine–thyroid interactions and to outline strategies for improving diagnosis, management, and prevention of thyroid dysfunction in the elderly.

## 2. Materials and Methods

This work was conceived as a narrative review synthesizing current evidence on the relationship between iodine intake, thyroid dysfunction, and ageing-related modifiers, with a particular emphasis on the role of gut microbiota, nutrient status, pharmacologic exposures, and metabolic ageing. Though this is a narrative review, we used a structured search strategy to ensure broad coverage of the subject.

### 2.1. A Comprehensive Literature Search

A comprehensive literature search was conducted between January and June 2025 using PubMed/MEDLINE, Scopus, Web of Science, and Embase databases. Given the narrative nature of this review, peer-reviewed publications published between 1980 and 2025 were considered in order to capture both foundational and contemporary evidence relevant to iodine–thyroid interactions in ageing. Priority was given to studies involving older populations (≥65 years); when evidence from younger or mixed-age cohorts was cited, it was used only to support mechanistic concepts and is explicitly interpreted in the context of ageing. Search terms combined controlled vocabulary [MeSH/Emtree] and free-text keywords including “*iodine*”, “*thyroid*”, “*hypothyroidism*”, “*thyroid autoimmunity*”, “*ageing*”, “*elderly*”, “*microbiota*”, “*dysbiosis*”, “*polypharmacy*”, “*amiodarone*”, “*iodinated contrast*”, “*levothyroxine absorption*”, and “*micronutrients*”. Boolean operators [AND/OR] and truncation were applied to broaden sensitivity. Reference lists of retrieved papers and relevant reviews were also manually screened to identify additional eligible studies.

### 2.2. Eligibility Criteria

We included peer-reviewed publications in English from 1980 to 2025 that investigated the following:

- The impact of iodine on thyroid function in adults ≥60 years,
- Age-specific physiological changes in thyroid hormone regulation,
- Gut microbiota interactions with thyroid autoimmunity or hormone metabolism,
- Nutrient co-factors relevant to thyroid function (selenium, zinc, and iron),
- Pharmacological exposures (iodine-rich or thyroid-disrupting drugs, and absorption modifiers).

### 2.3. Exclusion Criteria

Exclusion criteria were non-human studies unless mechanistically informative, pediatric or pregnancy-focused studies, and conference abstracts without peer-reviewed full text.

### 2.4. Data Extraction and Synthesis

Data from eligible studies were extracted into evidence matrices and organized according to predefined themes: [i] iodine intake and thyroid physiology, [ii] ageing-related thyroid adaptations, [iii] Wolff–Chaikoff effect and autoregulatory failure, [iv] microbiota–thyroid axis, [v] nutritional and gastrointestinal changes, [vi] pharmacological and iatrogenic factors, and [vii] metabolic ageing. Findings were narratively synthesized and critically appraised, highlighting convergences, inconsistencies, and gaps in knowledge (Table 1). Given the narrative nature of the review, reference groupings reflect thematic contribution rather than exclusive categorization.

### 2.5. Quality Assessment

Given the heterogeneity of study designs (epidemiological surveys, clinical cohorts, mechanistic studies, and reviews), formal meta-analysis was not feasible. Instead, methodological rigor was evaluated qualitatively, emphasizing sample size, adjustment for confounders, and reproducibility. Where appropriate, higher-level evidence (systematic reviews, meta-analyses, and large cohort studies) was prioritized in interpretation.

## 3. Results and Discussions

### 3.1. Why Focus on Older Adults

Hypothyroidism is disproportionately prevalent in older adults, affecting approximately 8–12% of women and 3–6% of men over 65 years, much of it undiagnosed [29,30,31]. In the Atherosclerosis Risk in Communities (ARIC) study, populations over 65 had a higher prevalence of undiagnosed hypothyroidism (6.88%, from which 0.82% clinically manifest and 6.06% subclinical) [32]. Risk is amplified by age-related physiological and clinical factors. Thyroid gland reserve declines with age, TSH set-points shift upward, and the capacity to escape from the Wolff–Chaikoff effect is attenuated [33]. Some studies show that 7–14% of older individuals have serum TSH levels above the upper limit of reference ranges [24,34,35,36]. The rise in TSH is independent of the presence of antithyroid antibodies [37]. However, there are also studies that found decreased serum TSH in the elderly [38,39]. Frequently, an opposite relation between TSH and age was noticed in iodine-deficient populations where the main thyroid pathology consists of the presence of nodules and higher thyroid autoimmunity instances as people age [40].

Older adults also have higher prevalence of multimorbidity and are more frequently exposed to drugs that interfere with thyroid function or iodine metabolism. Beyond pharmacology, gastrointestinal changes may impair iodine absorption and modulate thyroid autoimmunity [41]. Moreover, renal function decline with ageing prolongs plasma iodide half-life, potentially enhancing the risk from high-iodine medical exposures.

Epidemiological evidence from iodine-replete countries shows that both deficiency- and excess-induced hypothyroidism disproportionately affect the elderly, reinforcing the need for targeted iodine surveillance in this population [23]. In the context of rapid global ageing, ensuring adequate—but not excessive—iodine intake in older adults is becoming a critical component of endocrine public health.

Another factor to consider is the reduced salt intake in older adults. In many settings, discretionary household salt is the dominant iodine vehicle; thus, sodium restriction—common in older adults with hypertension or chronic kidney disease—can reduce iodine intake if iodized table salt is simply removed without replacement. U.S. NHANES data showed that adults reporting low-salt diets had lower urinary iodine and higher odds of iodine deficiency, underscoring this linkage at the population level [42]. Prospective data in hypertensive outpatients (mean age ~64 years) indicate that sodium reduction does not jeopardize iodine status when the small amount of discretionary salt that remains is iodized; importantly, most processed/restaurant foods in that setting used non-iodized industrial salt, so table salt remained the key iodine source. These findings align with World Health Organization and the Iodine Global Network (IGN) guidance: salt-reduction and salt-iodization agendas are compatible if iodization extends to all edible salt [including industrial/food-service and any salt substitutes] and if programs monitor iodine status in at-risk groups (pregnant women and older adults) [43,44].

For older patients with chronic kidney disease (CKD), the picture is doubly nuanced; they are often counseled to restrict sodium, which may lower iodine intake, yet reduced renal clearance alters iodine kinetics and can heighten susceptibility to iodine-induced dysfunction after large iatrogenic loads—arguing for individualized monitoring rather than blanket assumptions about adequacy.

Epidemiological data directly comparing thyroid disorders in older adults across different iodine nutrition settings remain limited, but available evidence supports a U-shaped relationship between iodine intake and thyroid dysfunction at all ages, including the elderly. Populations transitioning from iodine deficiency to sufficiency often experience transient increases in autoimmune thyroiditis, subclinical hypothyroidism, and iodine-induced hyperthyroidism, particularly among older adults with pre-existing nodular thyroid disease. Conversely, in iodine-replete or high-exposure regions, excess iodine more commonly precipitates hypothyroidism through impaired autoregulation, while hyperthyroidism occurs less frequently. Large cohort studies such as NHANES, ARIC, EPIC-Norfolk, HIMS, and HUNT have consistently shown that thyroid dysfunction increases with age, though the interpretation of thyroid tests must account for physiological shifts in TSH distribution (see Section 3.2.1) Accordingly, the use of age-specific reference ranges for thyroid function tests has been increasingly recommended to avoid overdiagnosis and overtreatment of subclinical hypothyroidism in the elderly.

Together, these features make older adults a high-risk group for both deficiency- and excess-related hypothyroidism, even in countries with established iodine prophylaxis. This intersection of epidemiology, physiology, and pharmacology justifies targeted monitoring and tailored clinical management strategies in ageing populations.

### 3.2. Age-Related Thyroid Physiology and Iodine-Related Pathophysiology

#### 3.2.1. Age-Specific Thyroid Physiology

Less investigated, age specific changes are noted in thyroid physiology. Thus, TSH shifts with ageing. Large epidemiological datasets (NHANES, EPIC, HIMS, and HUNT) show that serum TSH concentrations tend to increase modestly with chronological age, even in individuals without overt thyroid disease [45,46,47,48]. This rightward shift of the reference distribution appears most pronounced after the seventh decade, with median TSH values rising by ~0.5–1.0 mIU/L compared with middle-aged adults.

Some explanatory mechanisms might include

- Altered hypothalamic–pituitary set-point—possible reduced sensitivity of hypothalamic thyrotropin-releasing hormone (TRH) neurons and/or pituitary thyrotrophs to circulating free T4 [49].
- Reduced TSH bioactivity—some studies suggest changes in glycosylation patterns that may make TSH less biologically potent, thus requiring higher serum levels to achieve the same thyroidal stimulation [50].
- Adaptive or benign physiological change—higher TSH in elderly may reflect a homeostatic adaptation to slow metabolism and reduced tissue demand for thyroid hormones, not necessarily pathological hypothyroidism.

This age-related shift underlies the clinical debate about whether higher “normal” TSH ranges should be used in older adults, to avoid overdiagnosis and overtreatment of subclinical hypothyroidism [51]. Also with ageing, the thyroid gland undergoes structural and functional changes. They range from histological changes (increased fibrosis, decreased follicular cell mass, accumulation of colloid, and variable atrophy) and vascular and stromal alterations (microvascular rarefaction and reduced capillary density can impair hormone export) to autoimmunity accumulation (lifetime exposure to immune triggers increases the prevalence of anti-TPO (thyroid peroxidase) and anti-Tg antibodies, which can further reduce functional reserve) [52]. These changes lower the gland’s capacity to maintain euthyroidism under stress (e.g., illness, abrupt iodine load, and medication interference). As a result, older adults may be less able to compensate for environmental or pharmacologic disruptions in hormone synthesis. Throughout the manuscript, age-related physiological changes in thyroid function are distinguished from biochemical patterns observed in treated hypothyroid patients receiving levothyroxine, which are discussed separately where relevant.

**Figure 1:**
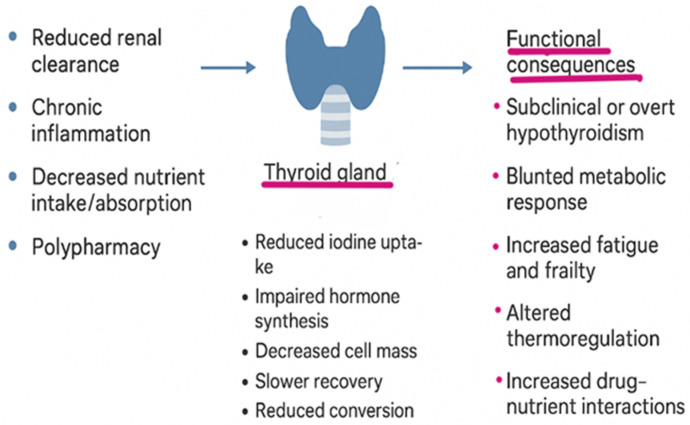
Age-related changes in iodine–thyroid physiology.

Collectively, these alterations render older adults more susceptible to both overt hypothyroidism and subtle thyroid dysfunction, even in the absence of frank disease (see Figure 1).

#### 3.2.2. The Wolff–Chaikoff Effect

The Wolff–Chaikoff effect is an acute autoregulatory response of the thyroid gland to excess intrathyroidal iodide, first described in 1948 [53]. When intrathyroidal iodide exceeds a critical threshold (~10^−3^–10^−2^ M), thyroid peroxidase (TPO)-mediated organification is transiently inhibited, leading to a rapid fall in monoiodotyrosine (MIT), diiodotyrosine (DIT), and thyroid hormone synthesis. In euthyroid individuals, this inhibition resolves within 24–48 h as downregulation of the sodium–iodide symporter (NIS) reduces iodide influx, restoring normal hormone synthesis. Failure to “escape” the effect—due to impaired NIS regulation, reduced follicular cell mass, or autoimmune injury—can result in sustained hypothyroidism. This occurs more frequently in neonates, individuals with autoimmune thyroiditis, and older adults, whose thyroidal reserve and adaptive capacity are diminished. In ageing, decreased efficiency of NIS downregulation, cellular signaling alterations, and coexisting autoimmunity may delay recovery after iodine excess. Comorbidities such as chronic kidney disease (CKD) prolong iodine retention, while high-iodine exposures further elevate risk [54,55,56]. Clinically, these mechanisms mean that even moderate iodine fluctuations can trigger subclinical or overt hypothyroidism in older individuals who would otherwise remain euthyroid. The effect therefore represents a central pathway linking iodine exposure to thyroid dysfunction in ageing populations and underscores the need for age-aware interpretation of thyroid tests and careful monitoring after iodine-rich intervention [49].

#### 3.2.3. The U-Shaped Curve: Deficiency and Excess as Dual Hazards

Population studies consistently demonstrate a U-shaped association between habitual iodine intake and the risk of thyroid dysfunction [25]. Chronic iodine deficiency (<100 µg/day in adults) impairs thyroid hormone synthesis, leading to elevated TSH, compensatory thyroid hypertrophy, and over time the development of nodular goiter and functional autonomy. In such settings, sudden or sustained increases in iodine intake may precipitate iodine-induced hyperthyroidism (Jod–Basedow phenomenon), particularly in older adults with long-standing nodular disease. Conversely, chronic iodine excess (>300–500 µg/day) can trigger sustained inhibition of organification (failure to escape the Wolff–Chaikoff effect) and promote thyroid autoimmunity, particularly in genetically predisposed individuals [26]. This risk is mediated through increased iodination of thyroglobulin, enhanced oxidative stress within thyrocytes, and unmasking of latent autoimmune susceptibility [27]. Epidemiologic observations during and after salt-iodization programs in formerly deficient regions have documented transient rises in autoimmune thyroiditis and subclinical hypothyroidism, particularly in women [28]. While these trends stabilize over time, they underscore the narrow margin between deficiency and excess, and the importance of gradual, carefully monitored iodization strategies. In older adults, who already carry a higher background prevalence of thyroid autoantibodies, even modest excess exposure may accelerate progression to clinical hypothyroidism.

### 3.3. Modifiers of Iodine Status and Thyroid Function in Ageing

#### 3.3.1. Gut Physiology and Microbiota

Evidence linking gut microbiota alterations to thyroid dysfunction in older adults is emerging but remains preliminary, with most data derived from observational studies or indirect mechanistic models. Taken together, current evidence supports a modulatory—rather than causal—role of the gut microbiota in iodine-related thyroid vulnerability in seniors. In this context, gut microbiota may exert multidimensional effects on thyroid function through immune modulation, nutrient metabolism, and hormone recirculation. Age-related dysbiosis may contribute to increased susceptibility to hypothyroidism and autoimmunity, while targeted interventions with probiotics, prebiotics, and diet hold potential but require rigorous clinical evaluation. Emerging evidence indicates that gut microbiota may play a pivotal role in modulating thyroid function, particularly in older adults, in whom age-related changes in microbiota composition may lead to an increased risk of thyroid malfunction [57]. The interactions are multifactorial, encompassing immune modulation, micronutrient metabolism, enterohepatic circulation, and peripheral thyroid hormone metabolism. These effects are mediated through microbial metabolites, cytokine modulation, and mucosal barrier function [58,59].

- Immune Modulation and Thyroid Autoimmunity

The gut microbiota exerts profound effects on systemic immune responses, shaping both innate and adaptive immunity. Dysbiosis—characterized by reduced microbial diversity and expansion of pathobionts—can promote pro-inflammatory signaling and the breakdown of immune tolerance. In older adults, this contributes to the increased prevalence of autoimmune thyroid diseases (AITD), including Hashimoto’s thyroiditis [60]. Microbial metabolites, such as short-chain fatty acids (SCFAs), regulate T cells (Tregs) and influence the production of cytokines that modulate thyroid autoantibody formation (anti-thyroid peroxidase (anti-TPO) and anti-thyroglobulin (anti-Tg) [61]. These metabolites act through G-protein-coupled receptors (e.g., FFAR2/3), modulate gene expression epigenetically, and thereby regulate immune cell behavior [62]. Experimental models suggest that certain bacterial taxa, including Bacteroides and Clostridia clusters IV and XIVa, enhance Treg differentiation, whereas reduced abundance may promote autoimmunity [63,64].

- Microbial Effects on Micronutrient Metabolism

Gut bacteria influence the absorption and bioavailability of micronutrients [65] including those critical for thyroid function [66]. Dysbiosis can impair selenium absorption and alter microbial metabolism, indirectly affecting thyroid hormone production and oxidative stress in the gland [67]. Similarly, microbial deiodinase-like activity might influence local iodine utilization and thyroid hormone synthesis, suggesting that gut composition may impact endocrine homeostasis [68,69].

- Influence on Enterohepatic Circulation and Peripheral Thyroid Hormone Metabolism

Thyroid hormones (T4, T3) are conjugated in the liver (glucuronidation/sulfation) and excreted into bile; a portion is deconjugated in the intestine by microbial β-glucuronidases and sulfatases, allowing enterohepatic recirculation and reabsorption of free iodothyronines. This enterohepatic loop has been shown in classic animal and human work (biliary secretion of iodothyronines; deconjugation by intestinal bacteria; interruption by bile acid sequestrants), and contemporary reviews explicitly recognize the microbiota’s role via these deconjugating enzymes [69]. Direct modulation of T4→T3 conversion by the microbiota has not been demonstrated; T4→T3 activation is mediated by host deiodinases, though microbiota can indirectly influence peripheral hormone availability by altering deconjugation and bile acid metabolism [70].

**Figure 2:**
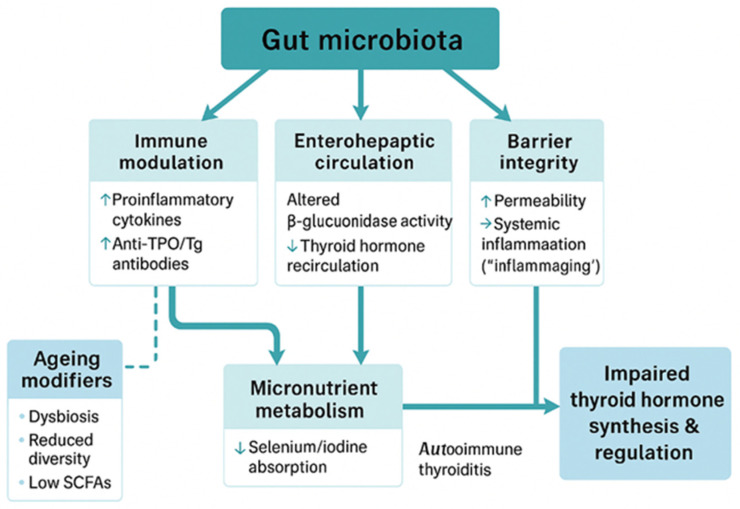
Proposed mechanisms linking gut microbiota and thyroid function in ageing. (TPO/Tg antibodies = thyroid peroxidase/thyroglobulin antibodies; SCF = short-chain fatty acid).

Dysbiosis in older adults, characterized by reduced microbial diversity and altered enzymatic activity, may impair β-glucuronidase-mediated deconjugation of thyroid hormone metabolites, thereby modifying the efficiency of enterohepatic recirculation. This could reduce peripheral hormone availability and subtly influence systemic thyroid status [71]. Although microbiota do not catalyze the T4→T3 conversion directly, changes in microbial composition may shape hormone reabsorption and metabolic outcomes, contributing to the variability observed in age-related subclinical hypothyroidism. The key mechanisms proposed to link gut microbiota and thyroid function in ageing—encompassing immune modulation, micronutrient metabolism, enterohepatic recirculation, and intestinal barrier integrity—are summarized schematically in Figure 2.

- Age-Related Microbiota Changes

Aging is accompanied by profound alterations in gut microbial ecology. Multiple cohort studies demonstrate that older adults typically exhibit reduced α-diversity and a decline in beneficial commensals, particularly butyrate-producing Firmicutes alongside enrichment of potentially pro-inflammatory taxa such as Enterobacteriaceae and other Proteobacteria [105].

**Figure 3:**
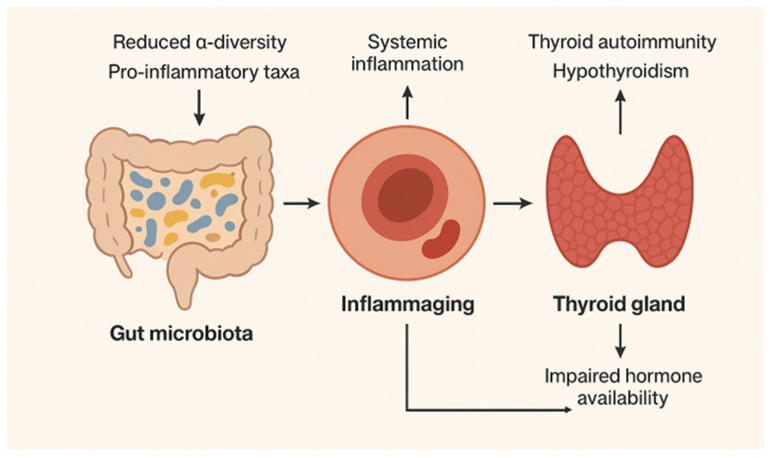
Microbiota, inflammation and thyroid functioning.

These compositional changes are not merely incidental but are closely linked with systemic inflammatory tone. Reduced short-chain fatty acid (SCFA) production and increased lipopolysaccharide (LPS) exposure contribute to gut barrier dysfunction, endotoxemia, and the phenomenon termed “inflammaging”—a state of chronic low-grade inflammation associated with impaired immune regulation [106] (see Figure 3). This pro-inflammatory environment has downstream relevance to the thyroid. Chronic inflammation can promote loss of immune tolerance, favoring thyroid autoimmunity [107]. Moreover, dysbiosis-driven alterations in bile acid metabolism and β-glucuronidase activity may influence enterohepatic recirculation of thyroid hormones, subtly modulating peripheral hormone availability. While direct clinical links between aging microbiota and hypothyroidism are not fully established, the biological plausibility is strengthened by these converging mechanisms.

Epidemiologic work has highlighted the variability within older populations. For example, frail elderly populations often present with pronounced loss of diversity and expansion of Proteobacteria, correlating with elevated systemic inflammatory markers and poorer metabolic outcomes [107]. In contrast, centenarians—despite advanced chronological age—frequently retain greater microbial richness, with enrichment of taxa such as Akkermansia and certain SCFA producers [108]. These individuals tend to exhibit lower levels of systemic inflammation and autoantibody prevalence, suggesting a protective role of preserved microbial diversity in buffering against immune dysregulation and possibly thyroid dysfunction. The findings are consistent with a protective phenotype and the survivor bias has to be taken into consideration. Thus, age-related microbiota changes can be conceptualized not as a uniform decline but as a spectrum ranging from resilience to dysbiosis. Individuals maintaining balanced microbial ecosystems may experience less inflammaging and lower risk of thyroid autoimmunity, whereas those with pronounced dysbiosis could face compounding risks from chronic inflammation, micronutrient malabsorption, and impaired enterohepatic recirculation of thyroid hormones. These results underscore the need for longitudinal studies that integrate microbiome, endocrine, and immune parameters in older adults to clarify causal pathways and intervention opportunities.

Taken together, current evidence supports a modulatory—rather than causal—role of the gut microbiota in iodine-related thyroid vulnerability in older adults.

- Potential of Probiotics, Prebiotics, and Symbiotics in Modulating Thyroid Outcomes

Interventional studies using probiotics, prebiotics, and synbiotics in thyroid disorders remain limited but encouraging. Meta-analyses indicate that probiotics can reduce systemic inflammatory markers [109], and randomized trials suggest improvements in intestinal barrier function [58] (e.g., lower zonulin). In Graves’ disease, in a small trial, adjunctive Bifidobacterium longum with methimazole improved thyroid function and autoantibodies, supporting a gut–thyroid axis mechanism [110]. While Lactobacillus rhamnosus strains consistently modulate inflammation and barrier integrity direct effects on thyroid hormone metabolism are better documented in animal studies with Lactobacillus reuteri [111] rather than Lactobacillus rhamnosus; thus, strain-specific claims should be made cautiously pending larger human trials. Clinical trials in older adults with thyroid disorders are scarce, and evidence remains preliminary [112]. In recent research [113,114,115], there have been some mixed effects reported of prescribing routinely prebiotic, probiotic, and synbiotic supplementation on clinical and laboratory outcomes in hypo- and hyperthyroidism.

#### 3.3.2. Changes in Dietary Habits, Nutrition, and Gastrointestinal Physiology

Aging is accompanied by profound alterations in dietary intake, gastrointestinal structure and function, and nutrient absorption [72], all of which bear relevance for thyroid health and iodine metabolism.

- **Dietary Transitions in Aging**

Nutritional patterns in older populations diverge from those of younger adults due to multiple interdependent factors. Physiological changes in appetite regulation (e.g., blunted ghrelin signaling, and increased cholecystokinin) combine with psychosocial determinants (loneliness, depression, and economic hardship), leading to lower caloric and nutrient intake [73,74]. Lower caloric intake is often accompanied by reduced protein and micronutrient density, increasing the risk of borderline deficiencies even in high-income settings. Older adults often consume less dairy and seafood [75], which are major iodine contributors in many countries, partly due to lactose intolerance, dental problems, cost, and personal preferences. At the same time, increased reliance on processed foods in institutionalized or socially vulnerable elderly may uncouple iodine intake from natural sources, depending on fortification policies [76]. Another factor that may play a role is salt use variability. Concerns about hypertension and cardiovascular disease often drive reduced discretionary salt use in seniors. When iodized salt is the main prophylaxis vehicle, this may inadvertently compromise iodine status unless compensated by fortified foods or supplements [77]. Supplement intake is inconsistent in older populations: some seniors adopt multivitamins or kelp-based iodine supplements [16], while others avoid supplements altogether. This produces wide inter-individual variability, with risks of both mild chronic deficiency and iatrogenic excess. Importantly, seniors are more likely to consume over-the-counter supplements without medical guidance, which can destabilize thyroid homeostasis.

- **Gastrointestinal (GI) Structural and Functional Alterations**

The GI tract undergoes age-related modifications that influence nutrient assimilation.

Gastric function: Hypochlorhydria and atrophic gastritis are prevalent in aging, compounded by frequent proton pump inhibitor (PPI) use. Low gastric acidity reduces iodine liberation from organic food matrices, impairs solubilization of iron and zinc, and alters microbiota composition [78]. Impaired gastric intrinsic factor secretion also predisposes to vitamin B_12_ deficiency, which may indirectly influence thyroid symptoms through overlapping neurocognitive manifestations.

Pancreatic and biliary function: Exocrine pancreatic insufficiency, though mild in many older adults, impairs lipid digestion and absorption of fat-soluble vitamins (A, D, E, and K) [79], as well as selenium. Changes in bile acid composition and reduced pool size alter enterohepatic circulation of hormones, with potential implications for thyroid hormone metabolism.

Intestinal absorption: While gross small-intestinal morphology is often preserved with aging, subtle functional changes occur as follows: reduced expression of certain nutrient transporters, increased mucosal permeability, and higher prevalence of microscopic enteropathy [80]. These changes may contribute to reduced efficiency of iodine, selenium, and zinc absorption

Colonic function and microbiota: Slowed colonic transit and constipation alter the gut microbial milieu, influencing bile acid metabolism and short-chain fatty acid production. Dysbiosis, as discussed in previous sections, can secondarily affect enterohepatic recirculation of thyroid hormones and systemic inflammation, thus linking gastrointestinal physiology to thyroid outcomes in the elderly.

Consequences of nutrient absorption: Normally absorbed rapidly in the stomach and duodenum, iodine uptake may be compromised in hypochlorhydria and reduced gastric surface area. Even mild impairments can become clinically relevant when combined with lower intake. Selenium deficiency diminishes activity of deiodinases (responsible for T4-to-T3 conversion) and glutathione peroxidases, exacerbating oxidative stress within thyrocytes. Zinc deficiency, more common in institutionalized elderly, alters thyroid hormone receptor activity and impairs TSH signaling [81]. These micronutrient deficiencies often coexist with impaired gastrointestinal absorption, creating a cumulative burden on thyroid function. In frail elderly, low protein intake reduces hepatic synthesis of thyroxine-binding globulin [82]. This alters the distribution and availability of thyroid hormones, complicating the interpretation of thyroid function tests and sometimes mimicking hypothyroid biochemistry.

#### 3.3.3. Pharmacologic and Iatrogenic Factors Affecting Thyroid Function in Older Adults

Thyroid function in older adults is particularly vulnerable to pharmacologic and iatrogenic influences due to age-related physiological changes, polypharmacy, and comorbidities. A systematic approach involving regular monitoring, judicious drug selection, and attention to drug–nutrient interactions is critical for preserving thyroid health in the aging population. Several drugs can directly or indirectly alter thyroid hormone synthesis, metabolism, or bioavailability, thereby affecting clinical outcomes.

- **Iodine-Rich Drugs**

Excess iodine can disrupt thyroid function through the Wolff–Chaikoff effect, a rapid autoregulatory block of hormone synthesis; failure to escape this inhibition may lead to prolonged hypothyroidism in susceptible older adults or those with autoimmune disease [53]. Ingesting high iodine loads—such as via iodinated contrast media used in imaging—can precipitate iodine-induced hyperthyroidism (Jod–Basedow phenomenon) or hypothyroidism [87]. The risk of iodine-induced hyperthyroidism is particularly high in older adults from iodine-deficient or previously deficient regions with nodular thyroid disease. Amiodarone, a potent I¬containing antiarrhythmic (~37% by weight), interferes with peripheral deiodination (inhibiting type I 5′-deiodinase), reduces T4→T3 conversion, and increases reverse T3, leading to various thyroid dysfunctions. It may induce hypothyroidism (AIH) via the Wolff–Chaikoff effect—particularly in iodine-replete settings—or hyperthyroidism (AIT) in patients with multinodular or latent thyroid disease, risks that increase with age and underlying glandular autonomy [54,88,89].

- **Thyroid-Toxic Drugs**

Certain drugs can directly injure thyroid tissue or trigger autoimmune thyroiditis. Used in psychiatric disorders, lithium inhibits thyroid hormone release, leading to hypothyroidism in up to 20% of patients, with higher susceptibility in older adults [90]. Lithium can also cause goiter via increased TSH stimulation [91]. Immune Checkpoint Inhibitors (ICIs) (Anti-PD-1, anti-PD-L1, and anti-CTLA-4) therapies used in oncology can trigger thyroiditis through immune activation, resulting in transient thyrotoxicosis followed by hypothyroidism. Incidence rises with combination regimens, and older patients may present with atypical or subclinical manifestations [92]. Tyrosine Kinase Inhibitors (TKIs), such as sunitinib or sorafenib, may reduce thyroid hormone synthesis, increase hormone clearance, or cause destructive thyroiditis. Older patients may be particularly sensitive due to reduced thyroid reserve [93].

- **Drugs Interfering with Levothyroxine Absorption**

Optimal thyroid hormone replacement can be impaired by numerous agents through direct chelation, pH alteration, or bile acid interactions [94]. Calcium and iron supplements form insoluble complexes with levothyroxine, reducing absorption. Proton Pump Inhibitors (PPIs) rise gastric pH, thus decreasing levothyroxine dissolution and absorption. Bile acid binders like cholestyramine and colestipol sequester levothyroxine in the gut. High fiber, soy products, or certain proteins can slow gastrointestinal transit or bind thyroid hormone, impairing bioavailability [95]. Careful timing of levothyroxine administration relative to these agents is essential, typically spacing doses by 2–4 h.

- **Polypharmacy Patterns and Cumulative Impact in Older Adults**

Older adults frequently take multiple medications for cardiovascular, metabolic, and psychiatric conditions. Polypharmacy can amplify the risk of thyroid dysfunction [96] through additive effects of iodine-rich or thyroid-toxic drugs. Also, there might be multiple agents impairing levothyroxine absorption [97] or drug–drug interactions affecting hepatic metabolism of thyroid hormones (e.g., CYP450 inducers such as rifampicin). We also have to consider the whole framework of metabolic changes, with increased susceptibility to subclinical hypothyroidism or thyrotoxicosis due to reduced thyroid reserve and altered homeostatic responses with aging. While polypharmacy is highly prevalent in older adults and creates multiple paths for pharmacologic and absorption interference with thyroid hormone management, epidemiological evidence directly linking polypharmacy to abnormal TSH or free T4 levels in community populations is lacking. Nevertheless, clinical guidelines recommend cautious, individualized monitoring of thyroid function in elderly patients due to heightened risk of drug interactions and altered hormone metabolism.

- **Clinical Implications and Management**

As a consequence, regular thyroid function testing is recommended in older adults on amiodarone, lithium, ICIs, TKIs, or high-dose iodine exposure. Minimizing unnecessary polypharmacy and carefully timing levothyroxine relative to interfering agents. Awareness of age-specific pharmacokinetics and comorbidities can guide drug selection and dosing to reduce thyroid-related complications.

#### 3.3.4. Metabolic Ageing and Thyroid–Iodine Interaction

Ageing is accompanied by profound alterations in metabolic homeostasis that influence thyroid physiology and the utilization of iodine. Beyond changes in intake and renal excretion, intrinsic metabolic shifts modulate hormone synthesis, conversion, and tissue responsiveness. Metabolic ageing creates a permissive environment where insulin resistance, adipokine imbalance, inflammaging, and micronutrient insufficiencies converge to affect thyroid hormone production, peripheral metabolism, and iodine utilization. This multifactorial landscape underscores the need for individualized nutritional and metabolic assessment in geriatric thyroid care. Also, while metabolic syndrome is not iodine-specific, its high prevalence in older populations and its interaction with inflammatory and metabolic pathways justify its consideration as a contextual modifier of iodine–thyroid interactions in ageing.

- **Chronic Low-Grade Inflammation (“Inflammaging”)**

Immune modulation and thyroid autoimmunity are important topics in older people. Age-related chronic low-grade inflammation—characterized by elevated IL-6, TNF-α, and CRP—can influence thyroid autoimmunity by promoting antigen presentation and autoreactive lymphocyte activation, subtly increasing anti-TPO or anti-Tg prevalence [98]. Inflammaging may impair thyroidal iodine uptake through oxidative stress and altered sodium–iodide symporter expression, potentially reducing hormone synthesis efficiency, particularly under borderline iodine intake [99,100,101].

- **Influence of renal function decline on iodine excretion**

After midlife, the glomerular filtration rate (GFR) declines by ~0.75–1 mL/min/1.73 m^2^ per year, even in the absence of overt kidney disease [102]. Because >90% of dietary iodine is excreted renally, this progressive decline leads to longer systemic retention of iodine in older adults, amplifying susceptibility to iodine-induced hypothyroidism (failure to escape the Wolff–Chaikoff effect) or hyperthyroidism (especially in nodular thyroids). Older adults often present with reduced thyroidal functional reserve (due to atrophy, fibrosis, and nodular autonomy), meaning that modest delays in iodine clearance can have disproportionate endocrine effects. Clinical studies show that elderly populations exposed to iodinated contrast media or amiodarone exhibit higher rates of thyroid dysfunction than younger cohorts with similar exposures [103]. This indicates a synergism between age-related renal and thyroidal changes. Even without chronic kidney disease, common geriatric factors—such as reduced protein and dairy intake, variable use of iodized salt, and lower fluid intake—alter iodine intake and excretion dynamics [104]. In parallel, polypharmacy (e.g., diuretics, ACE inhibitors, and NSAIDs) can subtly reduce renal clearance.

- **Micronutrient Co-Factors in Metabolic Ageing**

Beyond iodine itself, several micronutrients modulate thyroid hormone synthesis and metabolism and may influence iodine-related thyroid vulnerability in older adults. Selenium is essential for deiodinase enzymes and antioxidant selenoproteins that protect the thyroid from oxidative stress. Selenium deficiency reduces selenoprotein activity (e.g., glutathione peroxidases and deiodinases), thereby potentiating iodine-induced oxidative damage in thyrocytes [83]. Older adults often exhibit borderline selenium deficiency due to dietary limitations and reduced absorption, exacerbating thyroid vulnerability under inflammatory conditions [91]. Zinc is required for TRH gene expression and for the activity of type 1 deiodinase, which converts T4 into active T3. In humans, zinc deficiency has been associated with low T3 and elevated reverse T3, and supplementation restored thyroid function in small clinical studies [84]. Older adults are particularly vulnerable to zinc insufficiency due to reduced intake and absorption, making this an important factor in age-related thyroid dysregulation [85]. Iron is a co-factor for thyroid peroxidase (TPO), the enzyme responsible for iodide oxidation and thyroglobulin iodination. Iron deficiency reduces TPO activity and thyroid hormone synthesis [86]. In both animals and humans, iron deficiency leads to lower T4 and T3 concentrations; repletion restores normal thyroid function [86]. Subclinical iron deficiency, common in older adults due to dietary changes, comorbidities and polypharmacy, may thus contribute to subtle hypothyroid states despite adequate iodine intake by limiting effective iodide organification. These micronutrient deficiencies, frequently encountered in older adults, may therefore modify the clinical expression of iodine-related thyroid dysfunction rather than acting as independent causes.

### 3.4. Clinical Implications and Management Strategies in Older Adults

**Figure 4:**
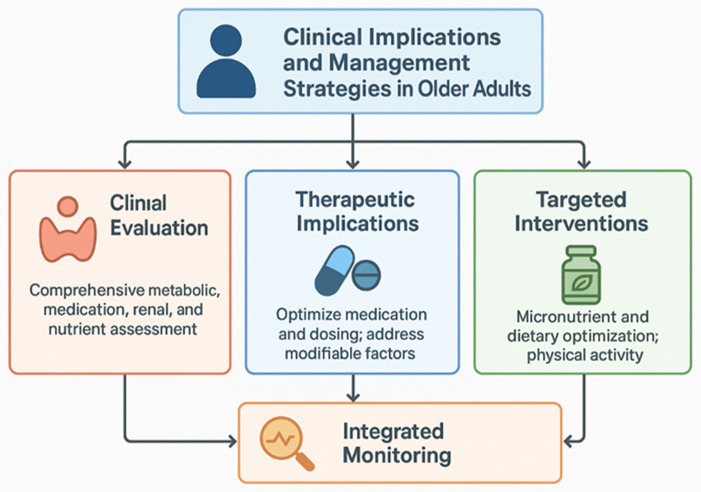
Older adults require a special integrated clinical management in order to ensure an adequate functioning of the thyroid.

Older adults are exposed to multiple, often synergistic factors that influence thyroid homeostasis. These factors can independently or jointly modify thyroid hormone synthesis, metabolism, and tissue responsiveness, and they require careful clinical interpretation (Figure 4). Older adults frequently present with higher TSH and lower free T3 even in the absence of overt disease [116], underscoring the need to avoid reflexive overtreatment.

Comprehensive evaluation should include metabolic profile, medication history, renal function, micronutrient status, and autoimmune markers to correctly interpret thyroid test abnormalities. Fluctuations in thyroid function may occur with acute illness, nutritional variation, and medication changes [117].

For patients on levothyroxine, dosing should consider reduced lean body mass, variable absorption, and interactions with calcium, iron, and fiber supplements; adjusting timing or dividing doses may improve absorption. Correcting modifiable contributors—such as micronutrient insufficiencies, dysbiosis, and metabolic derangements—may enhance thyroid function without escalating pharmacologic therapy [118]. In individuals with elevated anti-TPO or anti-Tg antibodies, periodic surveillance is warranted, particularly in the context of metabolic syndrome or inflammaging. Adequate intake of iodine, selenium, zinc, and iron should be ensured preferably via diet, with supplementation reserved for confirmed deficiencies [119]. Microbiome-targeted interventions, including probiotics, prebiotics, or dietary modulation, may complement traditional management, although robust evidence in older adults remains limited [59].

### 3.5. Limitations

This narrative review did not follow a systematic protocol, and therefore publication bias or incomplete retrieval of relevant studies cannot be excluded. The evidence base itself is heterogeneous, spanning epidemiological surveys, clinical cohorts, mechanistic models, and reviews of variable methodological quality; this precluded quantitative synthesis. Moreover, many data on iodine status, thyroid dysfunction, and related modifiers derive from younger or mixed-age populations, limiting direct applicability to adults ≥ 60 years. Evidence on the gut microbiota–thyroid axis is also preliminary, with few interventional studies in older adults. Finally, interactions among polypharmacy, nutrient status, renal function, and thyroid physiology remain complex and incompletely characterized, which constrains the strength of some inferences.

## 4. Conclusions

In older adults, thyroid function is particularly sensitive to variations in iodine intake, with iodine deficiency playing an important role in the development of hypothyroidism, while excess iodine exposure acts as a less frequent but clinically relevant destabilizing factor. Interpretation of thyroid function requires careful distinction between physiological ageing, particularly in the very old, and treated hypothyroid states, as iodine-related vulnerability may differ substantially between these conditions. Nutritional status, polypharmacy, and other age-related modifiers may further influence thyroid homeostasis, largely through their interaction with iodine metabolism and thyroid autoregulation. In this population, the risks of overtreatment—particularly cardiovascular complications, bone loss, and iatrogenic hyperthyroidism—frequently outweigh potential benefits. Management should therefore prioritize individualized decision-making that considers frailty, functional status, cognitive trajectory, and patient goals. For most subclinical abnormalities, a conservative strategy with periodic re-evaluation is appropriate, while clear indications for treatment should be approached with cautious dose titration. Continued research is needed to refine age-specific reference ranges and to better define long-term outcomes, but current evidence supports a balanced, context-driven approach that minimizes harm and maintains quality of life in older adults. Maintaining adequate and age-appropriate iodine intake should therefore remain a central consideration in the prevention and interpretation of thyroid dysfunction in older adults.

## Figures and Tables

**Table 1:** **Studies included in** **the narrative review**.

Topic	References	Notes
Iodine intake and thyroid physiology	[1,2,3,4,5,6,7,8,9,10,11,12,13,14,15,16,17,18,19,20,21,22,23,24,25,26,27,28]	Core iodine biology, deficiency/excess, population data
Ageing-related thyroid adaptations	[3,4,29,30,31,32,33,34,35,36,37,38,39,40,41,42,43,44,45,46,47,48,49,50,51,52]	TSH shifts, glandular reserve, ageing physiology
Wolff–Chaikoff effect and autoregulatory failure	[25,26,27,28,53,54,55,56]	Iodine-induced dysfunction, escape failure
Microbiota–thyroid axis	[57,58,59,60,61,62,63,64,65,66,67,68,69,70,71]	Immune modulation, enterohepatic circulation; evidence limited
Nutritional and gastrointestinal changes	[72,73,74,75,76,77,78,79,80,81,82,83,84,85,86]	Intake, absorption, micronutrient co-factors
Pharmacological and iatrogenic factors	[53,54,55,56,87,88,89,90,91,92,93,94,95,96,97]	Amiodarone, lithium, PPIs, immune-checkpoint inhibitors, contrast agents, medications affecting levothyroxine absorption
Metabolic ageing and comorbidities	[98,99,100,101,102,103,104]	Inflammaging, renal clearance, metabolic context

## Data Availability

No new data were created or analyzed in this study.
